# Pronounced Impact of *p*-Type Carriers and Reduction of Bandgap in Semiconducting ZnTe Thin Films by Cu Doping for Intermediate Buffer Layer in Heterojunction Solar Cells

**DOI:** 10.3390/ma12081359

**Published:** 2019-04-25

**Authors:** Waqar Mahmood, Saif Ullah Awan, Amad Ud Din, Junaid Ali, Muhammad Farooq Nasir, Nazakat Ali, Anwar ul Haq, Muhammad Kamran, Bushra Parveen, Muhammad Rafiq, Nazar Abbas Shah

**Affiliations:** 1 Material Synthesis & Characterizations (MSC) Laboratory, Department of Physics, Fatima Jinnah Women University (FJWU), The Mall Rawalpindi 46000, Pakistan; 2 Photon Science Institute (PSI), School of Physics and Astronomy, University of Manchester, Oxford Road, Manchester M13 9PL, UK; 3 Thin Films Technology (TFT) Research Laboratory, Department of Physics, COMSATS University Islamabad (CUI), Islamabad 44000, Pakistan; waqar00923335167800@gmail.com; 4 Department of Electrical Engineering, NUST College of Electrical and Mechanical Engineering, National University of Science and Technology (NUST), Islamabad 44000, Pakistan; ullahphy@gmail.com; 5 Analog Electronics System (AES) Laboratory, Department of Physics, Fatima Jinnah Women University (FJWU), The Mall Rawalpindi 46000, Pakistan; ammad@fjwu.edu.pk; 6 Department of Physics, COMSATS University Islamabad (CUI), Islamabad 44000, Pakistan; junaid_ali@comsats.edu.pk (J.A.); kamrankhattak@comsats.edu.pk (M.K.); 7 Department of Physics, RIPHAH International University, Islamabad 44000, Pakistan; farooq.nasir@riphah.edu.pk; 8 Department of Materials Science & Engineering, Institute of Space Technology (IST), Islamabad Highway, Islamabad 44000, Pakistan; nazakat@ist.edu.pk; 9 Department of Physics, Govt. Postgraduate College (Boys) Satellite Town, Rawalpindi 46000, Pakistan; anwaar60@yahoo.com; 10 Department of Physics, Lahore Garrison University, Lahore 54000, Pakistan; bushra.physics@lgu.edu.pk; 11 Department of Mathematics, COMSATS University Islamabad (CUI) Wah Campus, Wah Cantt 47040, Pakistan; rafiq@ciitwah.edu.pk

**Keywords:** semiconductor thin films, CSS, ion-exchange, XRD, SEM, *p*-type carriers, band gap, solar cell

## Abstract

Stabilized un-doped Zinc Telluride (ZnTe) thin films were grown on glass substrates under vacuum using a closed space sublimation (CSS) technique. A dilute copper nitrate solution (0.1/100 mL) was prepared for copper doping, known as an ion exchange process, in the matrix of the ZnTe thin film. The reproducible polycrystalline cubic structure of undoped and the Cu doped ZnTe thin films with preferred orientation (111) was confirmed by X-rays diffraction (XRD) technique. Lattice parameter analyses verified the expansion of unit cell volume after incorporation of Cu species into ZnTe thin films samples. The micrographs of scanning electron microscopy (SEM) were used to measure the variation in crystal sizes of samples. The energy dispersive X-rays were used to validate the elemental composition of undoped and Cu-doped ZnTe thin films. The bandgap energy 2.24 eV of the ZnTe thin film decreased after doping Cu to 2.20 eV and may be due to the introduction of acceptors states near to valance band. Optical studies showed that refractive index was measured from 2.18 to 3.24, whereas thicknesses varied between 220 nm to 320 nm for un-doped and Cu doped ZnTe thin film, respectively, using the Swanepoel model. The oxidation states of Zn^+2^, Te^+2^, and Cu^+1^ through high resolution X-ray photoelectron spectroscopy (XPS) analyses was observed. The resistivity of thin films changed from ~10^7^ Ω·cm or undoped ZnTe to ~1 Ω·cm for Cu-doped ZnTe thin film, whereas *p*-type carrier concentration increased from 4 × 10^9^ cm^−2^ to 1.4 × 10^11^ cm^−2^, respectively. These results predicted that Cu-doped ZnTe thin film can be used as an ideal, efficient, and stable intermediate layer between metallic and absorber back contact for the heterojunction thin film solar cell technology.

## 1. Introduction

Cadmium telluride (CdTe) based heterojunction tandem solar cells require a heavily doped, *p*-type back contact material of low resistivity as a buffer layer capable of long term stability [1]. Simultaneously both high efficiency and stability of low cost photovoltaic cells are a challenging issue in solar cell technology. A thin film of ZnTe was used as a stable back contact in CdTe based solar cells due to its small valence band offset between ZnTe and CdTe of 0.05 eV [2]. Among II-VI semiconductors, ZnTe semiconductor is a naturally low carrier *p*-type system having direct band gap energy of 2.24 eV at room temperature, and an absorption coefficient of 10^5^ cm^−1^ [3]. Thin films of ZnTe can absorb visible light without any phonon assisted mechanisms, it is an attractive choice for electro-optic and opto-electronic applications in the visible spectral range [3,4]. ZnTe can play an ideal role as a stable and efficient intermediate layer with CdTe absorber and metallic back contact for heterojunction type solar cells technology. Experimentally, ZnTe semiconductor has proven itself as a low resistive, stable, and efficient back contact for polycrystalline ZnTe/CdTe/CdS/ITO solar cells [5]. Previously, the values of electron affinity and work function of *p*-type CdTe thin films has been reported as 4.28 eV and 5.78 eV, respectively. Semiconductors readily form a Schottky barrier with metals. However, an ohmic contact is highly desirable and the key issue in the fabrication of CdTe based photovoltaic cells [2]. Such high work function as mentioned above is not possible for metals as back contacts. Therefore, ZnTe is the best candidate to fulfill these requirements and compatibility with the CdTe based tandem solar cells [6]. High resistivity of ZnTe system is another bottleneck, therefore, a heavily doped *p*-type element is required to uplift the conductivity in a ZnTe semiconductor [6,7]. Copper (Cu) can act as a suitable *p*-type acceptor to achieve this goal. In addition, due to the self-compensating effects of the ZnTe semiconductor, the *p*-type nature will remain the same after Cu doping. Based on previous studies, ZnTe is a potential candidate for solar cell application due to its ohmic contact with CdTe when doped with Cu.

Additionally, ZnTe semiconductors have found applications as cryogenic scintillators [8], opto-refractive, optical data processing [3,4] blue LEDs [9,10], and now as a background buffer layer in second generation solar cells. The possibility of self-compensation doping [11] by metallic dopants is one of the reasons for their extensive use in opto-electronics. Previously, ZnTe has been used in ternary compounds like MgZnTe, CdZnTe, and HgZnTe as hybrid semiconductors [12]. ZnTe growth has been well documented by electro-chemical deposition, two-source evaporation, sputtering [12], and closed space sublimation (CSS) technique [13,14,15,16,17]. The CSS technique is advantageous compared for competing coating methods to its smart utilization of materials, due to small substrate-source separations (5 mm). The physical properties of CSS deposited thin film can be enhanced by well controlled doping elements (e.g., Ag, Cu, and In etc.) through the ion-exchange process [18]. The conductivity of the un-doped ZnTe system is *p*-type usually due to Zn vacancy defects (V_Zn_) generation during thin film growth. The resistivity can be reduced by metal doping of group I with acceptor properties like Au, Li, Ag, and Cu etc. Previous reports on Cu doped ZnTe reflects that it is a good dopant due to its shallow acceptor defect [2]. These defects are responsible for high carrier concentration that is usually enough to narrow down the ZnTe and metal interface barrier within the tunneling regime. The Cu-doping probably increases the hole carrier concentration in ZnTe semiconductor, and therefore helps in the ohmic contact formation. Generally, if Cu replace Zn^2+^ species as Cu^+^ state the probability of inducing *p*-type charge carriers will be greater as compared to Cu^2+^ state.

There are only a few reports on Cu–doped ZnTe thin films, and none on the oxidation state of Cu dopants. The search for novel materials in our group led us to report the development of a light Cu-doped ZnTe thin film samples. We have extended our research by studying ZnTe films doped with low concentrations of Cu. In the current study, we report the optimized parameters for the synthesis of un-doped ZnTe thin film system with high hole concentration using CSS and ion-exchange method for Cu doping. We have studied the structural, morphology, elemental composition analysis, optical, electronic and electrical properties of un-doped and Cu-doped ZnTe thin film samples. The reduction of bandgap energy by introducing the shallow acceptor states while increasing the hole carrier concentration of ZnTe thin film with Cu doping may be a good semiconducting material for intermediate buffer layer in heterojunction solar cells technology. 

## 2. Experimental Procedure

**Figure 1:**
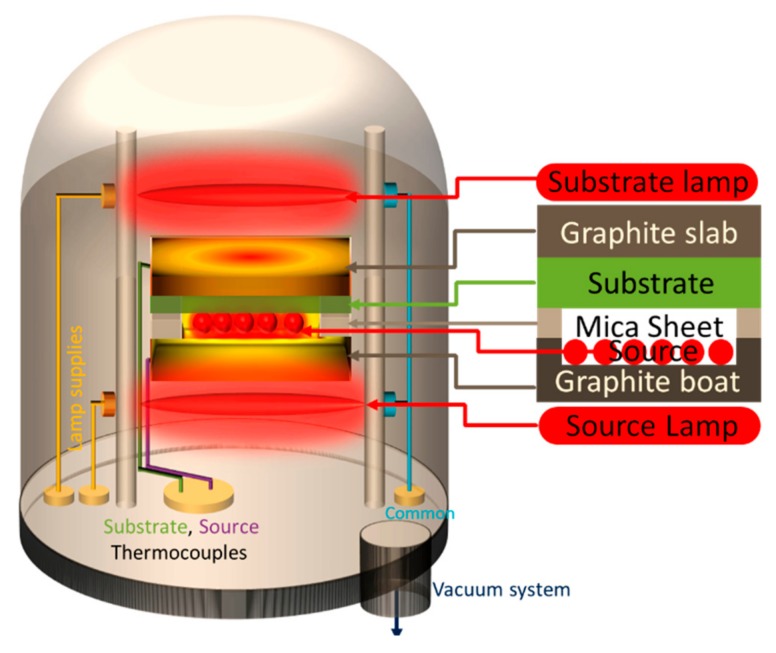
Schematic of Close Spaced Sublimation (CSS) technique used for deposition of ZnTe thin films.

Pure ZnTe powder (99.99% of Aldrich chemicals) was used as source material using a close spaced sublimation (CSS) technique to grow ZnTe thin films as shown in Figure 1. The source material was kept in a graphite boat, whereas glass slides were used as a substrate with graphite slab for uniform heating on the top of glass slide. The source-substrate distance was optimized to 5 mm for good quality of the films. The temperature gradient was created by the introduction of patterned mica sheet between the source and the substrate, as mica is a thermal insulator. Halogen lamps of 1000 Watts (500 Watts) were used to heat source (substrate). K-type thermocouples with temperature controllers were inserted into the graphite boat (slab) to monitor the temperatures of the source (substrate). The roughing (rotary) pump was used to achieve a pressure of 10^−2^ mbar. A diffusion pump was then employed to further decrease the pressure down to a vacuum level of 10^−5^ mbar. The halogen lamps were switched ON to gradually heat up, respectively, the source to 600 °C and the substrate to 450 °C. The source lamp was switched off by a stop watch after a deposition time (*t_d_* = 300 s), whereas the substrate lamp was kept ON for post deposition annealing. The chamber was left for cooling down to room temperature. A reddish brown ZnTe thin film was taken out from the chamber and tape test was done to check the film adherence. To obtain Cu-doped ZnTe thin film samples, un-doped ZnTe thin films were dipped in a low concentration copper nitrate Cu(NO_3_)_2_ solution in distilled water at (80 ± 5) °C. The doping was achieved and optimized by an immersion time of 40 min and drying the samples after immersion. An optimized post annealing process was achieved at (350 ± 5) °C for 1 h for the diffusion of Cu in ZnTe structure and to ensure the homogenous doping of Cu into ZnTe matrix.

The structural properties were recorded using a PANanalytical X’PERT PRO machine (Malvern Panalytical Ltd., Malvern, UK). The XRD patterns were recorded with operating conditions of 40 keV, 30 mA with Cu-Kα line (λ = 1.5406 Å). The scan speed was 1 s/step with 0.5° increments. An X-ray beam scanned the sample at angle, *θ*, from 20° to 60°. Scanning electron microscope (LSM-6490A analytical, JEOL, Tokyo, Japan) was used to study the surface morphology at electron beam energies 15–20 keV with 8 nm resolution and Energy Dispersive X-rays (EDX at 15 kV. The optical properties were measured using spectrophotometer of the Perkin Elmer LAMDA 950 with UV Win lab software in ultraviolet, visible, and near infra-red (UV-Vis-NIR) regions. The electrical measurements were taken under magnetic field 0.5 T, current 1 nA at 300 K using the Hall measurement system (HMS-5500 Ecopia, Ecopia Crop. Chandler Heights, AZ, USA). Cylindrical X-rays photoelectron spectroscopy (XPS) was adopted to investigate the bond strength and chemical oxidation states.

## 3. Results and Discussion

### 3.1. X-rays Diffraction (XRD)

**Figure 2:**
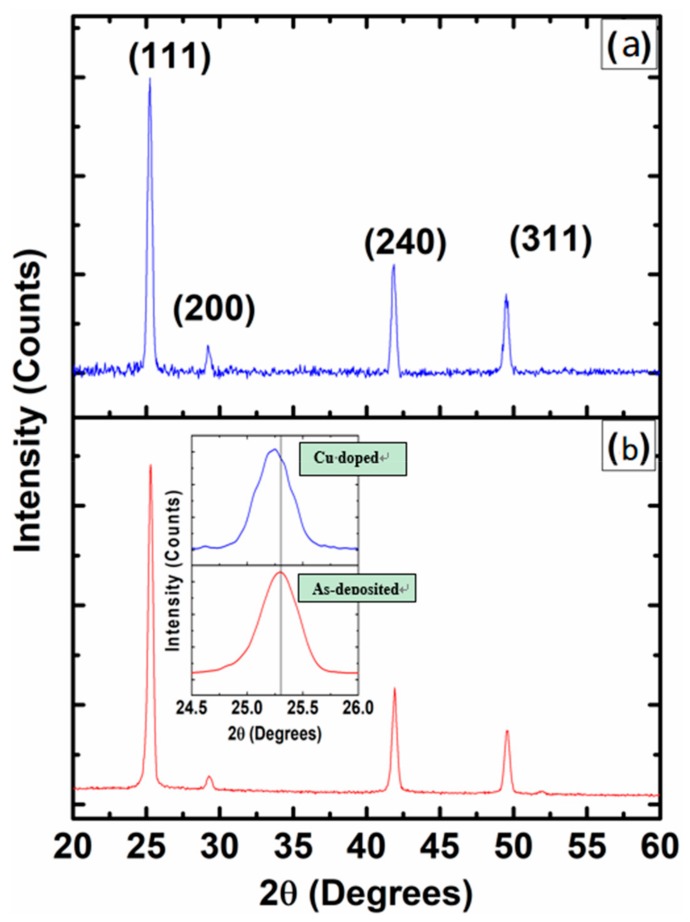
X-rays diffraction patterns of (**a**) un-doped ZnTe thin film and (**b**) Cu-doped ZnTe thin film samples. Inset shows the post-doping shift in (111) peak.

X-rays diffraction (XRD) measurements were performed for un-doped ZnTe thin films and Cu-doped ZnTe thin films samples to study the crystal structure. The level of intensity for un-doped ZnTe thin films was much higher compared to that of a Cu-doped ZnTe thin film samples. The decrease in intensity of the Cu-doped samples is induced by stresses produced in the ZnTe matrix due to Cu diffusion. The observed decrease of Bragg reflection intensity confirms the fact Cu is incorporated [18]. XRD data confirmed that (111) was the preferred orientation in ZnTe thin films and polycrystalline behavior with the cubic phase confirmed by International Card for Diffraction Data (ICDD) reference no. 00-089-3054 as shown in Figure 2. The peaks related to secondary phases or clustering issues of Cu-doped ZnTe system were not observed, which confirmed the presence of a single system of polycrystalline nature. After Cu doping, the XRD peaks shifted toward lower angle (2θ) values as shown in the inset of Figure 2. The lattice parameters are calculated for cubic unit cell and are found 6.09 Å and 6.12 Å for un-doped and Cu-doped ZnTe thin film samples, respectively. Overall, it was found that the unit cell volume increased as Cu incorporated into ZnTe thin films. As the atomic radius of Cu ~0.128 nm is larger than that of Zn ~0.074 nm [10], it was predicted that after doping Cu the unit cell volume will be enhanced, as we observed from the XRD data. Crystallite sizes of un-doped and Cu-doped ZnTe thin film samples were calculated using Scherer’s formula [10,19], Equation (1).

(1) $$\mathrm{Crystallite}\;\mathrm{Size}=\frac{0.9\lambda }{\beta \cos\theta }$$

where λ is the wavelength of X-rays, θ is the angle of diffraction and β is full width half maximum (FWHM). The crystallite size of un-doped ZnTe thin film was ~27 nm, which was increased to ~50 nm after immersion and diffusion of Cu into ZnTe samples for 40 min. Generally, cationic doping ions have a tendency to incorporate as interstitial (occupy voids space) site or substitute cationic species in semiconductors. Metallic Cu peaks or secondary phase peaks are not observed on the XRD diagram of Cu doped ZnTe layers. This suggests a Cu incorporation on substitutional sites. The possibility of Cu occupying at the interstitial site is very low, as the atomic size of Cu is larger than Zn species. Furthermore, we shall identify a single phase of samples from Raman spectroscopy and the oxidation state of Cu from XPS measurements along with the conductivity type from Hall measurement.

### 3.2. Raman Spectroscopy

**Figure 3:**
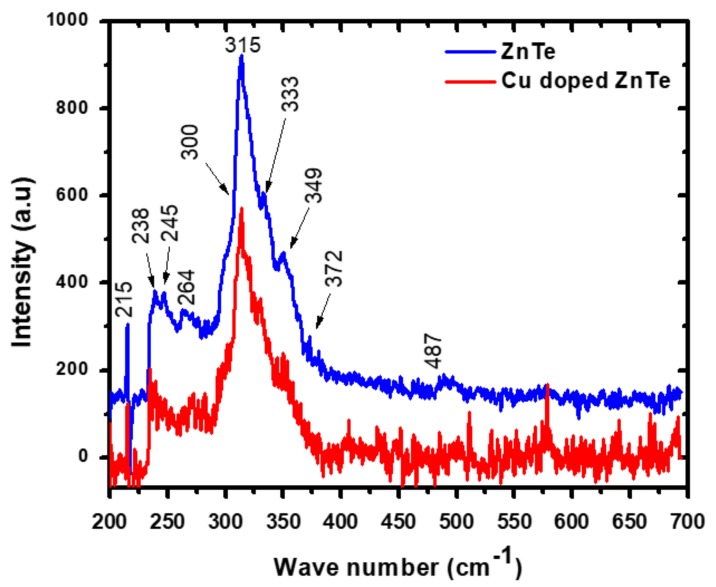
Room temperature Raman spectra of un-doped ZnTe and Cu-doped ZnTe thin film samples.

A non-destructive Raman spectroscopy technique was used to check the crystal structure of ZnTe thin films at room temperature (RT). Transverse (TO) and longitudinal (LO) modes were checked in first order Raman spectra. The upper vibrational frequency is expressed by LO and lower frequency is denoted by the TO mode [20]. RT-Raman spectroscopy measurements were performed and data of un-doped ZnTe thin films and Cu-doped ZnTe thin film samples have been presented in Figure 3. The intensity of the Raman peaks decreased after incorporating Cu species into ZnTe thin film samples as compared to un-doped ZnTe thin films. These results are correlated with the XRD results showing degradation in the crystallinity of ZnTe thin film sample with doping effects of Cu.

In addition, RT-Raman spectra related to the TO and LO modes at the Γ-point, numerous two-phonon characteristics correlating to dissimilar signals in the ZnTe semiconductor are observed. Four vibrational Raman modes were observed at low frequency region 200–290 cm^−1^ for both samples. The pronounced peak at position 215, and two bands 238 and 245 correspond to the vibration modes of multi-phonons L_O_-ZnTe, T_O_(X)+T_A_(X) and L_O_+T_A_(L), respectively, as calculated theoretically [20]. The Raman spectrum of both samples showed five multi-peaks under a large band in the range of 290 to 400 cm^−1^. The indication of each peak is very clear in the asymmetric band by kinks as shown in Figure 3. According to the Raman vibrational mode selection rules of ZnTe [20], it was observed, the peak at position 300 and 313 corresponds to 2L_A_(L) and T_O_(X) +L_A_(X) scattering modes. Similarly, L_O_(L) or T_O_(L)+L_A_(L) and L_O_(X)+T_O_(X) are observed at positions 333 and 349, respectively, in broad band as per selection rule system [20]. At the position 372 cm^−1^ a vibrational mode 2L_O_(X) has been identified in these spectra. No peak or band was found beyond 500 cm^−1^ i.e., high frequency region. It is noticed, a small shift towards lower frequency number of phonons was observed in the 1LO peak and 2LO peaks that may be due to disorder and strains introduced in ZnTe samples after Cu incorporation as reported in earlier studies [20,21,22].

Figure 3 showed that the most prominent band of un-doped ZnTe thin films lie centered at 315 cm^−1^, containing different phonon modes, was red-shifted to 313 cm^−1^ with incorporating Cu dopant in ZnTe thin film samples. This shift may be well corresponded to the structural results. In the XRD data, peaks are shifted towards a lower angle, similarly Raman band and peaks are also shifted toward lower wave numbers after incorporating Cu into the ZnTe matrix. Both the XRD and Raman characterizations, it may be suggested that the Cu dopant introduces substitutional defects at the cationic site, which could be responsible for generating higher stress in Cu-doped ZnTe thin film samples. In the XRD results, unit cell volume expanded as Cu diffused into the ZnTe structure, while the emission of phonon waves occurred at a lower frequency number after Cu doping and may strengthen the argument that Cu has properly replaced Zn species for substitutional defects. The lattice contraction has been observed by increasing the phonon frequency in the case of ZnTe bulk crystal system [20,21].

Furthermore, to better understand the effect of Cu doping in the ZnTe thin film, the concept of classical theory will be considered [21], given for LO and TO optical phonons by the following Equation (2).

(2) $$\omega LO\;\sim \;\omega TO\;\sim \;={\left(K/M_{e}\right)}^{1/2}$$

where, *K* is force constant and $M_{e}$ the extrinsic impurity atom’s mass.

Case 1: if $M_{e}<M$ (mass of the parent atom), the vibrational frequency will be increased, such that $\omega_{e}>\omega$. However, in case 2; if $M_{e}>M$ then $\omega_{e}<\omega$ and the frequency number of vibrational phonons will be reduced. Now in this case as the Cu substitutes the Zn species, the Zn species also remains within the interstitial sites of the stable cubic structure, thus the mass of Cu-doped ZnTe thin film sample is greater than un-doped ZnTe thin film. Thus, using the above equation approximation, case 2 is more relevant for these results. This is another mathematical reason that corresponds to the experimental results, where the Cu-doped ZnTe thin film samples the excited phonon frequency will be lower than the un-doped ZnTe sample as observed in RT-Raman spectra.

### 3.3. Microstructural Measurements

**Figure 4:**
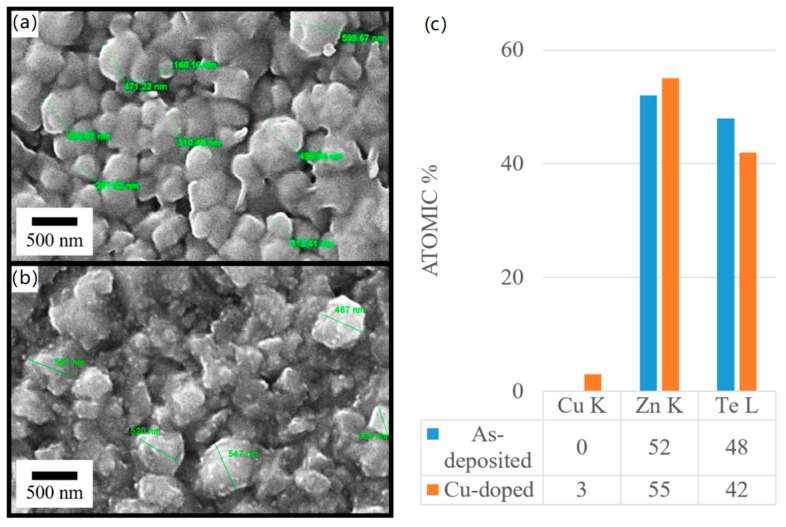
Scanning Electron Micrographs of (**a**) un-doped and (**b**) Cu-doped ZnTe thin film samples with (**c**) EDX analyses of un-doped and Cu-doped ZnTe thin film samples shows a Cu content of 3 at.% in Cu-doped samples.

Scanning electron microscopy (SEM) images of un-doped and Cu-doped ZnTe thin film samples has been demonstrated in Figure 4a,b. Elongated morphologies of crystals were observed, rather than proper circular, found in both un-doped and Cu-doped ZnTe thin film samples. Due to higher agglomeration effects in un-doped ZnTe thin films, it is very difficult to measure the exact grain size of all grains; however, we marked the crystals with their larger length only. In un-doped ZnTe thin films, the coalescence effect is more prominent, and we observed lager size crystals compared to Cu-doped ZnTe thin film samples. After incorporating Cu into ZnTe structure, the agglomeration effects reduced, and the clarity of the grains and grain boundaries were more visible and clearer in Cu-doped ZnTe thin film samples as compared to un-doped ZnTe thin films. In Cu-doped ZnTe thin film samples, due to local stresses induced by diffusing Cu atoms into the ZnTe grains may be a reason for the separation of agglomerated grains. Thus, from microstructural data, we may argue that Cu doping overall plays a vital role in the crystal for generating grain boundaries. A significant re-orientation and coalescence of the grains in other II-VI semiconductors are responsible for the larger crystals sizes reported earlier [13,18,23].

A tabulated result of EDX study was shown with Figure 4, emphasizing the change in elemental composition of un-doped ZnTe thin films and Cu-doped ZnTe thin film samples. Cu concentration increased within ZnTe grains and therefore it is evident that Cu substitutes Te, hence Te content decreased. In addition, it was redistributed, whereas the concentration of Zn seemed to increase after Cu immersion in ZnTe thin film samples. The highest reported efficiency was 3 at.% from 1 to 8 at.% in Tang et al. of Cu-doped ZnTe thin films [24].

### 3.4. Optical Transmission Spectroscopy

**Figure 5:**
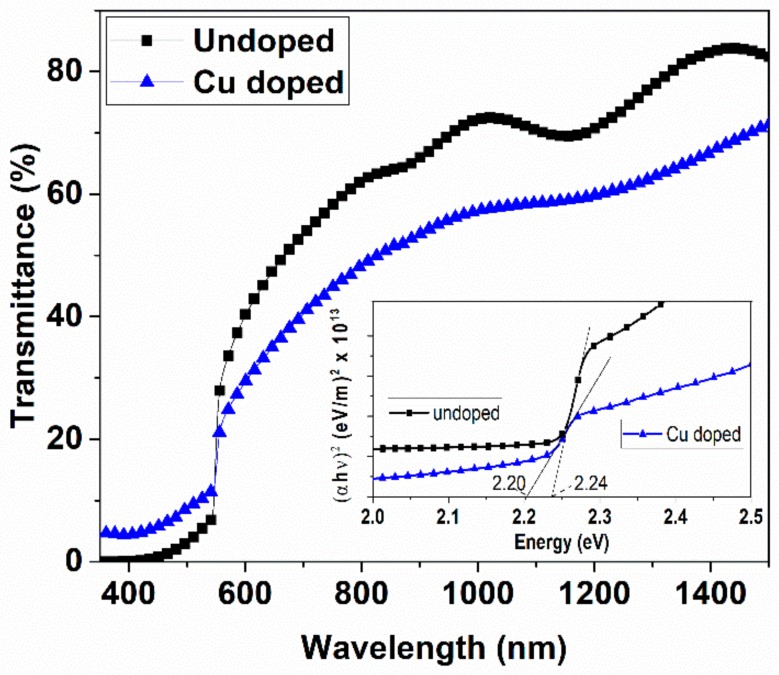
Transmission spectra of un-doped ZnTe thin films and Cu-doped ZnTe samples, the inset shows the calculation of bandgaps, using Tauc plots.

Optical spectroscopy data in transmission mode of Cu-doped and un-doped ZnTe thin film samples were shown in Figure 5. A decrease in transmission was observed in Cu-doped ZnTe thin film samples. The transmission of un-doped ZnTe thin films was 85%, which was decreased after Cu immersion down to 65%. The band gap energy was calculated by the absorption coefficient (α) as given in Equation (3) and the relation for Tauc plot given by Equation (4).

(3) $$\frac{1}{d}\ln\left(\frac{1}{T}\right)h\nu =\;\alpha$$

(4) $$\alpha .h\nu =A{\left(h\nu -E_{g}\right)}^{N/2}$$

where *A* is a constant, hυ is photon energy, $E_{g}$ is energy band gap of material [25].

Un-doped ZnTe thin film has a band gap of 2.24 eV, which was reduced to 2.20 eV, calculated through the above relations, due to Cu incorporation as shown in the inset of Figure 5. It was observed that the transmission and bandgap reduced for the Cu-doped in ZnTe thin film samples. The carrier concentration and grains of Cu-doped ZnTe thin film samples are responsible for band gap energy variation. The reduction of band gap energy indicates that the defect levels were introduced after Cu immersion into ZnTe thin films. It is predicted that as the Cu^+1^ incorporates into Zn^+2^, the possibility of hole carriers is obvious in Cu-doped ZnTe thin film samples. The Cu^+1^ dopant induces accepter states within the bandgap, close to the valance band, thus these acceptor states play a role for reducing the band gap energy in Cu-doped ZnTe thin film samples. The calculation of hole carrier concentration measurements will be correlated with these observed results. Similarly, variation of the band gap of un-doped ZnTe thin film was 2.24 eV reduced to 2.20 eV after Cu doping, as been reported earlier [10], but this group did not identify the role of oxidation state of Cu species. A theoretical model of Swanpoel was used to calculate the optical thickness of the thin films and refractive index by using the transmission data [26]. The refractive index *n* can be calculated by the Equation (5).

(5) $$n=\frac{N+\sqrt{N^{2}-4s^{2}}}{2}$$

where *N*, the number of oscillations, is related to the maximum transmission (*T_max_*) and minimum transmission (*T_min_*) as,

(6) $$N=1+s^{2}+4s\times \frac{T_{max}-T_{min}}{T_{max}\times T_{min}}$$

The thickness *d* of the target thin films can be calculated using the Equation (7) between wavelengths corresponding to maximum transmission (*λ_max_*) and minimum transmission (*λ_min_*).

(7) $$d=\frac{\lambda_{max}\;\times \;\lambda_{min}}{4n\left(\lambda_{max}-\lambda_{min}\right)}$$

Optical parameters like refractive index varies from 2.18 to 3.24 and optical thickness ranging from 320 nm to 220 nm were calculated using the above Equations (5)–(7) for un-doped and Cu-doped ZnTe thin film samples.

### 3.5. X-ray Photoelectron Spectroscopy (XPS)

X-rays photoelectron spectroscopy (XPS) has been employed to study the electronic structure and chemical oxidation states of the ZnTe thin films species along the elemental information of Cu after doping. The binding energy (B.E.) of photoelectrons at 284.5 eV was used as a charge referencing standard for C-1s peak. CASA-XPS software was used to de-convolute the high resolution XPS peaks of Zn, Te, and O species. Unfortunately, the Cu signal was not captured during Cu-2p high resolution measurement, which may be due to the very small amount of Cu doping concentration as confirmed by EDX. However, we identified Cu^1+^ peak in the high resolution Te-3d spectra as discussed below. The Lorentzian-Gaussian peaks were fitted on high resolution spectra of Zn, Te, and O nicely. All the fitted graphs have a non-linear Shirley type background and the other low-level elements were contained in baseline [17].

**Figure 6:**
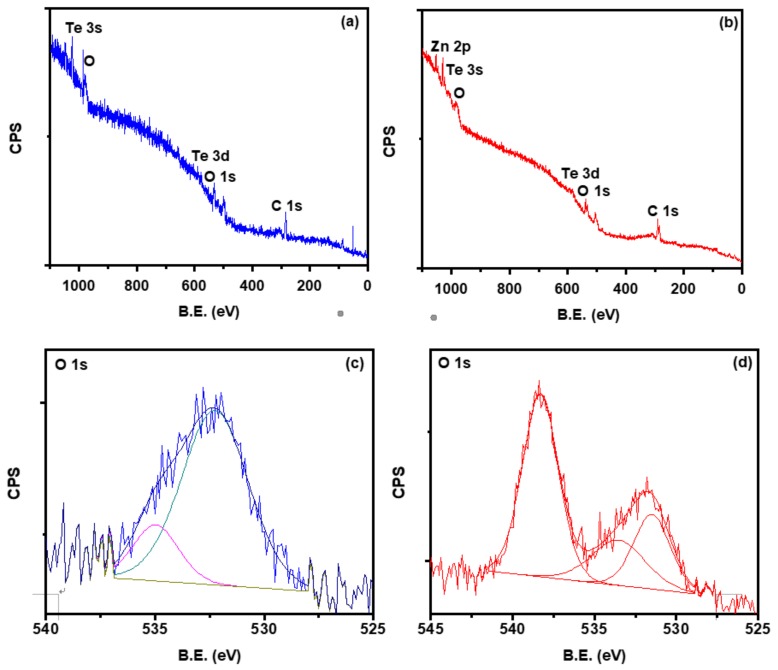
XPS Survey scan of (**a**) un-doped and (**b**) Cu-doped ZnTe thin film samples. De-convoluted XPS analysis of Oxygen-1s for (**c**) un-doped and (**d**) Cu-doped ZnTe thin film samples.

The survey scans of un-doped ZnTe and Cu-doped ZnTe thin film samples are presented in Figure 6a,b. The orbital presence of the fundamental elements of these sample’s zinc (Zn) and tellurium (Te) along oxygen (O) and carbon (C) has been observed. The Cu peak was not observed in the doped survey scan, which may be due to a lower percentage of doping. The signal of C-1s may be due to the presence of carbon tape used during the XPS measurements. The presence of an oxygen signal in the survey scan may be due to the surface contamination with environmental oxygen. It is further studied, the high resolution XPS measurements of all compositions individually.

High resolution XPS of O-1s peaks of un-doped and Cu-doped ZnTe thin film sample has been demonstrated in Figure 6c,d. The de-convoluted O-1s peak at 532.3eV and 535 eV in as deposited ZnTe thin films, were examined. The peak at 532.3 was due to –OH contamination on the surface, it might be due to chemisorption or dissociated oxygen [27]. The peak at 535 eV indicated the O–C species. The de-convoluted peak positions for O-1s after Cu doping in ZnTe thin films were observed at 531.50 eV, 533.6 eV, and 538.3eV [28]. The peak at 531.5 eV was due to concentration variation in oxygen vacancies of the deficient region. The chemisorption or OH species or dissociated oxygen was attributed at the peak of 533.6 eV, and the peak located at 538.3 eV might be due to the O–C contamination. The carbon or oxygen on the surface treated as contamination is the existence of auxiliary oxides formation. The level of intensities relates to the strong bonding between the species [17].

**Figure 7:**
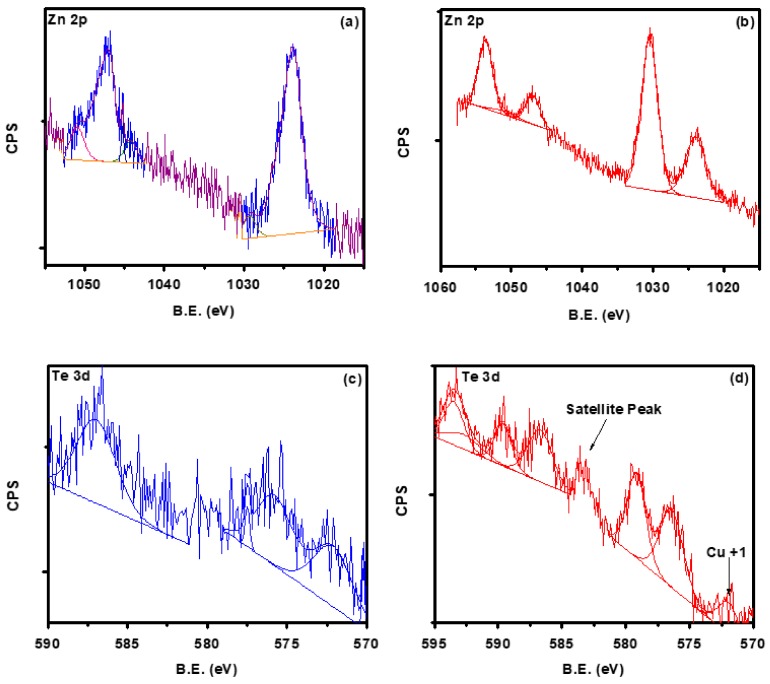
De-convoluted XPS analysis of Zinc-2p for (**a**) un-doped and (**b**) Cu-doped ZnTe thin film samples. De-convoluted XPS analysis of Tellurium-3d for (**c**) un-doped and (**d**) Cu-doped ZnTe thin film samples.

The high resolution XPS measurements were performed for Zn-2p spectra, while the de-convoluted fitted peaks for Zn-2p_3/2_ and Zn-2p_1/2_ of un-doped and Cu-doped ZnTe thin film samples are shown in Figure 7a,b, respectively. The intensity of Zn-2p_3/2_ peaks is usually higher, however, the binding energy (BE) is less than Zn-2p_1/2_ [22]. These fitted Zn-2p_3/2_ peaks of un-doped ZnTe thin films were observed at 1024.48 (due to Zn^2+^ oxidation) and 1023.63 eV (Zn metallic) while 2p_1/2_ lies at 1048.30 eV (due to Zn^2+^ oxidation) and 1046.84 eV (Zn metallic) peak central positions. The spin orbit coupling (ΔE = Zn-2p_1/2_ − Zn-2p_3/2_ = 23.82 eV) for un-doped ZnTe sample showing Zn^2+^ oxidation state is calculated. It is observed that in un-doped ZnTe thin films the 100% Zn-ions are not bonded with Te-ions. After doping of Cu in ZnTe matrix, the high resolution XPS Zn-2p peaks were shifted towards higher order values, mostly due to doping effects [12]. The higher intensity de-convoluted Zn-2p_3/2_ peak at position 1030.49 eV and Zn-2p_1/2_ at 1053.61 eV were obtained after fitting with ΔE = 23.12 eV that is showing the Zn^2+^ state. For Cu-doped ZnTe thin film samples the calculated Zn-2p_3/2_ peak at position 1023.86 eV and Zn-2p_1/2_ at eV 1047.16 are related to the un-bounded Zn species. The results may be inferred that with Cu doping, we observed less amount of Zn in metallic form as compared to Zn^2+^ state. In other words, Cu supports the introduction of more Zn^2+^ states in Cu-doped ZnTe thin film samples as compared to the un-doped ZnTe thin film.

High resolution XPS spectra after devolution of Te-3d for un-doped and Cu-doped samples have been presented in Figure 7c,d. The peak positions at 587.11 eV, 575.93 eV and 572.31 eV were observed for un-doped ZnTe thin films, whereas after Cu immersion the peaks were located at 593.55 eV, 589.69 eV, 586.74 eV, 579.17 eV, 576.50 eV, and 572.10 eV. The prominent, de-convoluted Te-3d_5/2_ and 3d_3/2_ peaks at positions (i.e., 587.46 and 576.96 for un-doped and for Cu doped 586.96 and 576.86) are observed, these are mostly due to oxide tellurium (TeO_2_, TeO_3_) as reported previously. Usually in the ZnTe system, the bonding between Te-Zn exists and can be observed during high resolution XPS spectra of Te-3d [7]. It is found that Te-3d_3/2_ was at peak position 572.73 eV for both samples, while we noticed Te-3d_5/2_ at peak position 583.16 eV for only the Cu-doped ZnTe thin film sample. However, in the literature mostly four peaks are observed in Te-3d spectra, while the observation was different for Te-3d of our un-doped and doped sample [2]. In un-doped ZnTe thin film, the XPS spectra peak of Te-3d_5/2_ was not present, which may indicate that a lower amount of Zn species were bonded with Te in the absence of Cu doping. Thus, here Cu plays a major role for enhancing the numbers of bonds between Te-Zn species. The Auger peaks were observed due to the incorporation of Cu into the matrix of ZnTe thin films. Another prominent peak at 569.87 eV were noticed and corresponded to Cu^+1^ species as reported in the literature [29,30]. Similarly, we found Zn *LMM* Auger peak [31] at position 579.60 eV in Cu-doped ZnTe thin film samples. These two Auger peaks were found only in Cu-doped sample and did not observe any indication of these signals in un-doped ZnTe thin films.

Overall, an interesting result was obtained from the electronic properties of both samples and there was clear indication that Cu^+1^ had been incorporated into the matrix of the ZnTe systems. There was no signal of Cu^2+^ observed in the XPS measurements. In this study, and in the observed results from the XPS data, the Cu^+1^ induced a larger number of hole carriers in the ZnTe thin film systems. For further verification of Cu^1+^, electrical measurements using the Hall setup performed to quantify the hole carrier concentration is discussed below. In our current study, the electron transport of ZnTe thin films doped with Cu have been observed with the identification of the oxidation state of Cu as Cu^1+^, while an earlier study did not focus on the oxidation state of Cu (i.e., Cu^1+^ or Cu^2+^) [12].

### 3.6. Electrical Properties

The Vander pauw technique [32] was employed to calculate the electron transport of Cu-doped and un-doped ZnTe thin film samples. The electrical reading was carried out five times at room temperature to ascertain them and minimize the errors. Un-doped ZnTe thin film possesses resistivity of the order of mega ohm-cm, which was reduced up to 1 ohm-cm after Cu immersion in matrix of ZnTe thin films. The Cu doping resulted in the reduction of resistivity due to increase in charge carriers in ZnTe thin film. The resistivity of un-doped ZnTe thin films was ~10^7^ Ω·cm that was reported by Maqsood et al. [33] decreased to ~ 0.5 Ω·cm after 40 min Cu diffusion into a ZnTe sample with concentrated solution. The mobility of un-doped ZnTe thin film was 16 cm^2^/Vs and increased to ~350 cm^2^/Vs for Cu immersed ZnTe thin film samples. The mobility increased after Cu-doping was due to the increase in the carrier concentration. The calculated sheet concentration for un-doped ZnTe thin film of ~4 × 10^9^/cm^2^ increased by two orders of magnitude to 1.4 × 10^11^/cm^2^ for 40 min Cu-doped ZnTe thin film samples. We observed the *p*-type conductivity in these Cu-doped ZnTe thin film samples. The substitution of Zn^+2^ by Cu^+1^ in doped samples can be obvious as the hole carrier concentration increased in the doped samples. These electrical results also support the chemical results that were measured by XPS and bandgap reduction in the transmission measurements.

## 4. Discussion and Conclusions

CdTe based solar cells are a potential candidate for large scale fabrication, however, low resistive back contact with p-type CdTe is still under attention. Mott-Schottky theory limits CdTe Fermi level pinning up to a certain extent. The acceptor compensation in CdTe with limiting effectiveness of contact and low doping efficiency that relies on quantum tunneling [34]. These constraints for forming good ohmic contact of CdTe like Au, Ag, or Cu. The intermediate layer should enable high p-type doping and small valence band offset with CdTe to get low resistance quantum mechanical tunneling. ZnTe is supposed to be a good candidate for such an intermediate layer. The small valence band offset for CdTe/ZnTe with Cu doping was introduced to >10^18^ cm^−3^ but the identification of Cu^+1^ or Cu^+2^ is not clear over there. The ZnTe contacts got reasonable success and stability after Cu immersion in solar cell devices. Cu is a quick diffuser in many materials [12]. Cu contents in semiconducting material were related to different instabilities in CdTe based solar cells. However, we focused on the investigation of Cu^+1^ diffusion into the matrix of ZnTe thin film system at optimized parameters, while reproducing the same carrier concentration repeatedly.

Thin films of ZnTe were synthesized at optimized parameters by sublimation through the CSS technique and Cu doping in ZnTe thin films—this was achieved using an ion exchange process. These samples showed a polycrystalline nature with (111) direction as a preferred orientation. The structural and morphological study correlated that as crystallite size increased, this resulted in the increase of grains of Cu-doped ZnTe thin film samples [13]. The elemental composition confirmed the Cu contents in ZnTe thin films using EDX. The transmission decreased from 85% to 65% after Cu immersion in optical properties. A slight decrease in band gap energy was observed after Cu immersion in ZnTe thin film samples. The resistivity decreased several order of magnitude (10^6^ to 1 Ω·cm) after Cu doping in electrical measurements. After doping Cu into the matrix of ZnTe, the unit cell volume increased as the ionic size of Cu species are larger than Zn species. The unit cell volume expansion may be related to the reduction of the bandgap. From the Hall measurements, the resistivity reduction of the ZnTe bare thin film as compared to Cu doped sample as the hole carrier increased due to the charge difference between the Cu^+1^ and Zn^+2^ species. These p-type charge carriers can play a vital role for the reduction of bandgap energy after incorporating Cu into ZnTe with the introduction of accepter states defects near to the valance band. These defects states are also identified from high resolution XPS studies of these samples. The XRD data confirmed the homogeneous distribution of Cu in ZnTe thin film samples. Unit cell volume increase confirmed the substitutional doping of Cu in crystal structure of ZnTe, while the XPS measurement confirmed the presence of Cu^+1^ oxidation state in ZnTe sample. These results support the picture and are correlated with electric measurements. Cu^+1^ has confirmed the substitution of Zn^+2^ species, thus the charge carrier’s concentration of *p*-type should be enhanced obviously as we found in electrical properties. Thus, at this stage our doped sample showed p-type conductivity in Cu^+1^ doped ZnTe thin film samples. The decreasing trend in resistivity of Cu-doped ZnTe thin film samples showed the semiconducting behavior.
